# Development and Verification of a Novel Robot-Integrated Fringe Projection 3D Scanning System for Large-Scale Metrology

**DOI:** 10.3390/s17122886

**Published:** 2017-12-12

**Authors:** Hui Du, Xiaobo Chen, Juntong Xi, Chengyi Yu, Bao Zhao

**Affiliations:** 1 School of Mechanical Engineering, Shanghai Jiao Tong University, Shanghai 200240, China; sdduhui@hotmail.com (H.D.); xiaoboc@sjtu.edu.cn (X.C.); jg5bvictor@sjtu.edu.cn (C.Y.); zhaobao1988@sjtu.edu.cn (B.Z.); 2 Shanghai Key Laboratory of Advanced Manufacturing Environment, Shanghai 200030, China; 3 State Key Laboratory of Mechanical System and Vibration, Shanghai Jiao Tong University, Shanghai 200240, China

**Keywords:** large-scale metrology, robot integrated system, structured light profilometry, hand-eye calibration, global data fusion

## Abstract

Large-scale surfaces are prevalent in advanced manufacturing industries, and 3D profilometry of these surfaces plays a pivotal role for quality control. This paper proposes a novel and flexible large-scale 3D scanning system assembled by combining a robot, a binocular structured light scanner and a laser tracker. The measurement principle and system construction of the integrated system are introduced. A mathematical model is established for the global data fusion. Subsequently, a robust method is introduced for the establishment of the end coordinate system. As for hand-eye calibration, the calibration ball is observed by the scanner and the laser tracker simultaneously. With this data, the hand-eye relationship is solved, and then an algorithm is built to get the transformation matrix between the end coordinate system and the world coordinate system. A validation experiment is designed to verify the proposed algorithms. Firstly, a hand-eye calibration experiment is implemented and the computation of the transformation matrix is done. Then a car body rear is measured 22 times in order to verify the global data fusion algorithm. The 3D shape of the rear is reconstructed successfully. To evaluate the precision of the proposed method, a metric tool is built and the results are presented.

## 1. Introduction

Large-scale thin wall and surface components are widespread in modern high-end manufacturing industries, especially in the automotive, shipbuilding, astronautical and aeronautical industry. In these fields, the surface forming quality usually equals the manufacturing quality of the corresponding component to some extent, and finally affects the assembly quality to a great degree. Bad performance in quality control means more pauses, modifications and even failures in the manufacturing process. This will undoubtedly lengthen the production cycle and the costs will increase for this reason. Therefore, to meet the precision requirements of manufacturing plants and reduce time/manpower costs, an automatic, flexible and accurate large-scale 3D measurement method is of great importance or even indispensable in some application scenarios.

Various methods have been developed in the field of large-scale 3D shape measurement and a lot of instruments have been introduced for this purpose [1,2,3,4]. Among off-the-shelf products, the Leica T-Scan has a good performance in large range metrology for its high speed and hand-held property [5]. However, it is not so suitable for on-site automatic inspection which is increasingly important in intelligent manufacturing. Traditionally, the coordinate measuring machine (CMM) has been extensively used in 3D shape measurement. For different products of different sizes, different CMMs are correspondingly developed. The measurement range spans from less than 1 m to several meters. With the development of visual sensor, computer vision technology and computation ability, more and more kinds of vision measurement equipment are integrated with CMMs, which are usually used in contact measurement for the dies, molds and so on [6,7,8]. However, the biggest drawback of this kind of method is that only limited types and numbers of products can be sampled and brought to the CMM for inspection. This means that even for relatively important products, it is difficult for us to obtain the quality data of all the components, which may lead to possible quality failure. If the CMM is integrated with the production line, it usually will not perform well in precision and robustness. Recently, with the improvement in precision manufacturing, robot kinematics and control engineering, robotic technologies have gone through huge developments. All these factors make robots increasingly economical and practical in manufacturing industry. Therefore, more and more visual sensors are integrated into robots to form a more flexible measurement system [9,10]. Furthermore, in some researches, a turntable surrounding the robot is introduced to extend the metrology range of the robot system [11,12]. Because it combines robot’s flexibility and accuracy of visual profilometry, it is promising for online inspection of large-scale parts. Another 3D shape measurement technology widely used in large volume profilometry is laser scanning [13,14,15]. The advantage of this kind of method is that it is easier to implement and more economical. Under the assumption of good calibration, the accuracy can also be assured. However, the weakness of this method is clear. In principle, laser scanning can only acquire data along one line or several lines for each measurement. To enhance the data quantity at every planned measurement position, a movement mechanism should be integrated with the scanner [16]. Normally a linear rail or a rotary mechanism is necessary to achieve this function. However, the introduction of the movement mechanism brings movement errors into the system. To compensate the errors, the movement mechanism should be calibrated, which is also a challenging task. Compared to the laser scanning method, structured light profilometry [17,18,19,20,21] can acquire the data on a surface for each measurement. Without the movement mechanism, the accuracy of every measurement depends only on the calibration of the visual sensors. If only the calibration is designed and implemented carefully, the accuracy can be assured [22,23]. Additionally, this method acquires much bigger data than laser scanning, which, compared to the line scanning method, produces more accurate metrology results. Meanwhile, thanks to the area scanning property, it has a better time performance. For all these advantages, the structured light scanning method will be promising if it is used in large-scale metrology. Some work has been made in this direction. Paoli et al. [24] mounted a binocular structured light scanner at the end of an anthropomorphic robot arm, and mounts the robot with two linear guides (horizontal and vertical). By building a series of coordinate systems, the measured data is unified into a fixed coordinate system defined by a total station. This approach works well in the measurement of a large yacht hull. However, as stated in Paoli’s paper, the position of the total station must be set carefully to ensure that all the optical reflectors can be observed simultaneously. To some extent, this limits the flexibility of the system.

Overall, compared to other technologies, structured light scanning is an accurate and efficient method for 3D metrology. The integration of structured light scanner and robot will dramatically enlarge the measurement volume. By appropriate hand-eye calibration and coordinate transformation, a software independent algorithm can be built, which makes large-scale data fusion a less challenging task. Until now, the study about this kind of system is limited. Therefore, more work should be done to improve the performance of this type of method.

In this paper, a novel integrated robotic scanning system is proposed for flexible and robust large-scale measurement. The system is composed of a binocular structured light scanner, a robot with six degrees of freedom (DOF) and a laser tracker. The structured light scanner is used to get the surface data in specific measurement positions. An optical target corner cube reflector (CCR) is fixed on the base of the scanner. The end coordinate system (ECS) is built by rotation of the scanner. The laser tracker is used to get the center of target ball and finish the data fusion. As for the hand-eye calibration, different from the traditional method, by observing the target ball using the scanner and the end coordinate system, the transformation matrix is computed. After obtaining the transformation between end coordinate system and world coordinate system (WCS), all the data is combined into the same coordinate system. In this way, the data fusion is finished automatically.

The rest of the paper is organized as follows: Section 2 introduces the overall measurement principle and system construction. The building of the end coordinate system and hand-eye calibration algorithm is also stated in this part. Section 3 introduces the results of hand-eye calibration and global data fusion. A quantitative evaluation is presented in this part. The paper will finish in Section 4 with a short conclusion.

## 2. The Proposed Approach

### 2.1. Measurement Model and System Construction

The integrated robotic scanning system incorporates an industrial robot with six degrees of freedom (DOF), a laser tracker, a digital fringe projecting (DFP) scanner and a CCR which is mounted on the scanner. The DFP scanner is fixed at the end effector of the robot. When the system works, the robot locates the scanner to the planned discrete positions, and the scanner acquires the 3D point cloud of that corresponding region.

**Figure 1:**
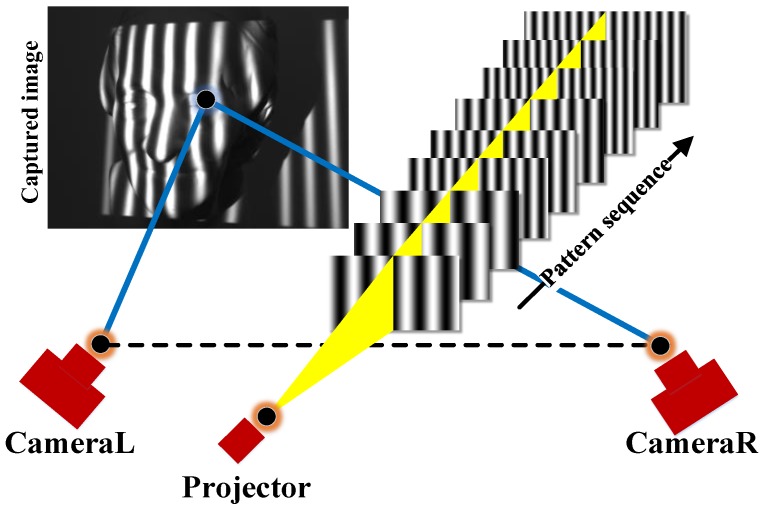
Measurement principle of the scanner.

Instead of choosing an off-the-shelf product, the scanner is a binocular DFP equipment developed according to the implementation circumstances (e.g., work distance, illumination and reflectivity of the surface). For its characteristics of high resolution and low sensitivity to the ambient light, the three-frequency and three-step phase shifting scheme is adopted to encode the fringe patterns. To access the data of a region, nine patterns are sequentially projected onto the surface by the projector. Then they are acquired by two cameras from different directions. After phase decoding and image registration, the final 3D point data is acquired. The working principle of the DFP scanner is illustrated in Figure 1.

Like any other vision metrology system, the cameras should be previously calibrated. To enhance the accuracy of calibration and measurement, a more accurate calibration method is applied [25]. This approach acquires the calibration points in a form of grid point array, and gets the calibration results by Zhang’s algorithm [26]. With this approach, high accuracy of calibration and measurement can be assured.

**Figure 2:**
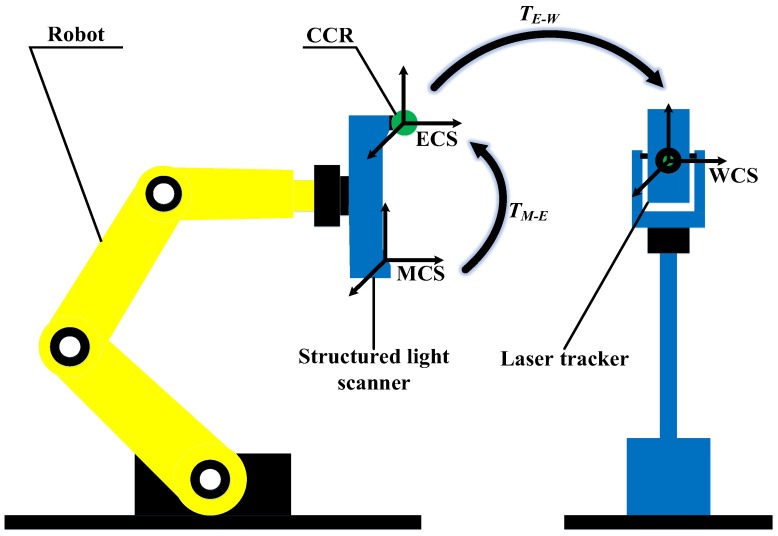
Definition of the coordinate system.

The coordinate system of the integrated system comprises measurement coordinate system (MCS), ECS and WCS, which is shown in Figure 2. The ECS is defined by the rotation of the scanner. The WCS is fixed with the laser tracker.

Being *P* a point in the robot workspace, the mapping relationship between coordinate *P_W_* in WCS and *P_M_* in MCS is expressed as follows:

(1) $$P_{W}=T_{E-W}T_{M-E}P_{M}$$

*T_M_*_−*E*_ is the transformation relationship between MCS and ECS, *T_E_*_−*W*_ denotes the transformation matrix between ECS and WCS. The acquired data of this integrated system is aligned and assessed in the world frame which is defined by the laser tracker.

By combining the area scanning structured light equipment, the laser tracker and the robot, this system reaches a high-level equilibrium in flexibility, velocity and accuracy. With the DFP scanner, 3D shape information of the object will be acquired at one robot position. Through off-line programming, the whole surface of the workpiece or specified features can be measured. In this case, the robot is only used to carry the scanner, and all the acquired data is unified to WCS. Compared to other methods, this scheme avoids the error accumulation of multiple coordinate transformation and robot main body calibration. Therefore, high accuracy could be expected. The adoption of area scanning ensures high efficiency and resolution of the system, which is crucial for subsequent data analysis. For a specified position, the scanning can be finished within 3 s, including the fringe projection time. With this integrated system, the complete measurement could be executed in a short period and the accuracy could be maintained.

**Figure 3:**
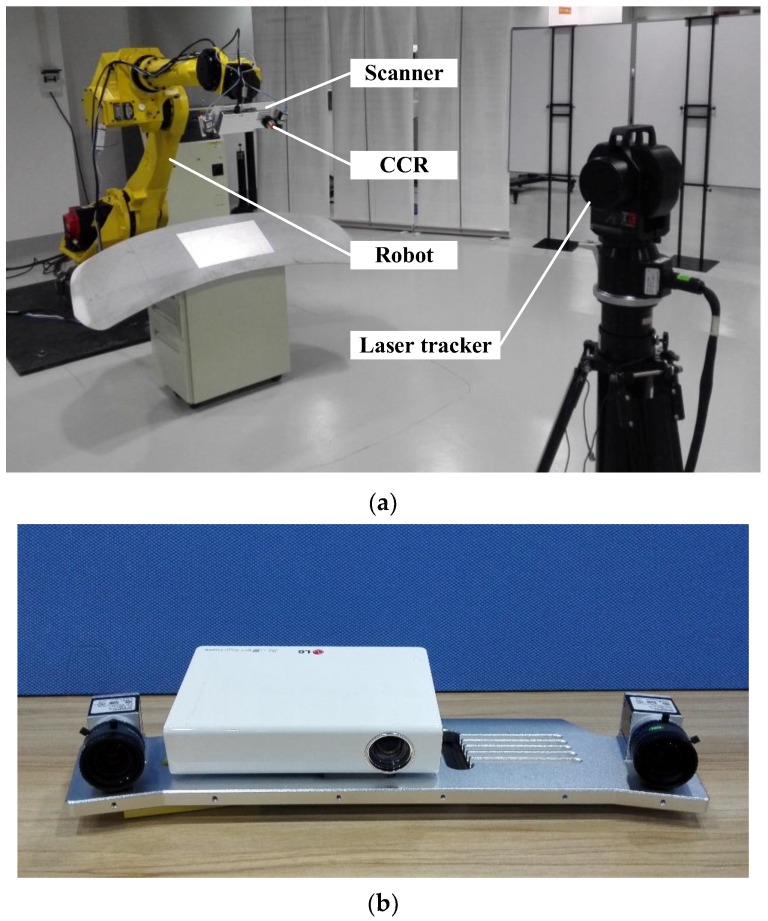
(**a**) Construction of the integrated 3D scanning system; (**b**) Binocular structured light scanner.

Mounting the scanner and CCR on the industrial robot, and putting the laser tracker API T3 in front of the six degree of freedom (DOF) Fanuc robot M-710iC, the integrated 3D scanning system is constructed as illustrated in Figure 3a. The construction of the structured light scanner is shown in Figure 3b.

As shown in Figure 3a, to construct the ECS, a CCR is set on the scanner. When the system works, the scanner acquires the point cloud first, and then rotates to another two positions. Using these three points, the ECS is built. Obviously, this method reduces the constraints of the relative position between the laser tracker and the reflectors. Actually, only one CCR is used in this method and the ECS is constructed by the rotation of the scanner. Therefore, if only the laser tracker is put at an appropriate position from the scanner, the ECS can be smoothly acquired by three times of rotation. Compared to other methods, this approach avoids most of the occlusion and is relatively more flexible and robust.

### 2.2. End Coordinate System Construction

**Figure 4:**
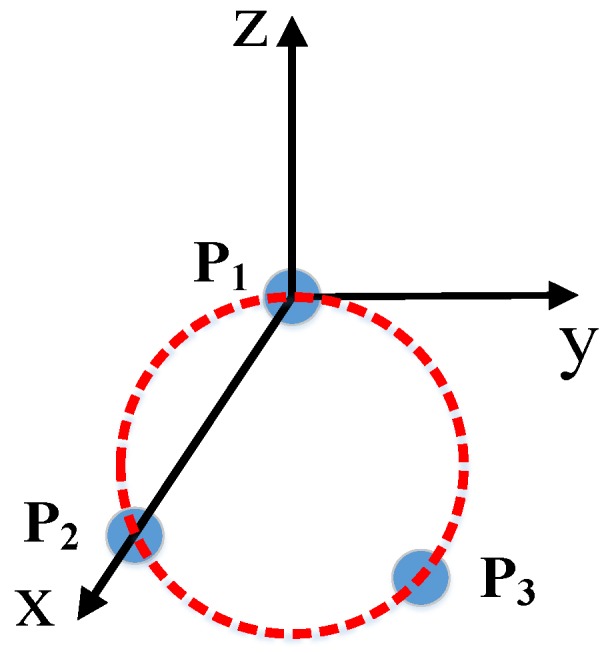
Construction of ECS.

As the beginning step of building the global data fusion algorithm, the ECS should be previously constructed. The robot’s J6 axis and the scanner are used to implement this work. The CCR is put on the basement of the scanner. When the system works, the scanner is positioned to a planned point and its position is acquired by the laser tracker. Then fringes are projected to acquire the point cloud of the corresponding surface area. After that, J6 axis rotates for another two times, and the position is recorded by the laser tracker. Finally, after rotation for three times, three points (P1, P2, P3) are recorded. The first point P1 can be taken as the origin point. Connecting P1 and P2 into a straight line, *X*-axis is in the same direction. *Z*-axis is built by vector cross product. *Y*-axis can be obtained by the same method. In this way, the ECS is constructed. This process is explained in Figure 4.

### 2.3. Hand-Eye Calibration

In classic hand-eye calibration algorithm, to acquire the hand-eye transformation matrix, the robot should take the eye to several different positions and observe the same calibration rig. The robot kinematics parameters are used to solve the transformation matrix. Different from the traditional method, in the proposed method, the robot is only used as an orienting device. It is unnecessary for us to apply robot’s kinematics parameters and the kinematics error can be bypassed. This benefits the improvement of calibration accuracy.

**Figure 5:**
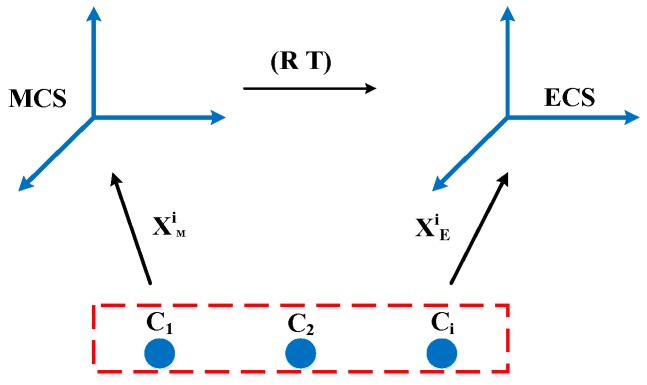
Hand-eye calibration.

In the proposed method, a CCR is used as the calibration target ball. In the calibration process, the ball is measured by the structured light scanner and laser tracker simultaneously (Figure 5). Firstly, to acquire the center of target ball, the scanner is used to get the point cloud data. This data is used to obtain the ball center in MCS, and this center can be denoted as $X_{M}^{i}$. For ease of use, it should be saved in the form of homogeneous coordinate. At the same time, the CCR ball is measured by the laser tracker. Actually, based on the principle of the laser tracker, the ball center can be acquired in this way. It can be denoted as *C_i_*. To transform *C_i_* into ECS, the ECS is first built as stated in Section 2.2. Taking P1 as the origin point, the coordinates can be acquired by projecting vector *P*_1_*C_i_* onto the three ECS axes. This new coordinate of *C_i_* can be denoted as $X_{E}^{i}$, which is also transformed to the homogeneous coordinate form. By putting the CCR at several different positions in the scanner vision field, two homogeneous coordinate vector groups are constructed, which are shown as follows:

(2) $$X_{M}=\left[X_{M}^{1}\;X_{M}^{2}\;X_{M}^{3}\;\cdots X_{M}^{i}\cdots \;\right]$$

(3) $$X_{E}\;=\left[X_{E}^{1}\;X_{E}^{2}\;X_{E}^{3}\;\cdots \;X_{E}^{i}\;\cdots \;\right]$$

To solve the transformation matrix between the scanner and ECS, an equation is built as follows:

(4) $$X_{M}=T_{E-M}X_{E}$$

where *T_E_*_−*M*_ is the hand-eye transformation matrix. It can be written into the following form:

(5) $$T_{E-M}={\left[\begin{array}{cc} R_{3\times 3} & T_{3\times 1}\\ 0_{1\times 3} & 1 \end{array}\right]}_{4\times 4}$$

In this matrix, *R* means the rotation matrix, and *T* the translation vector. According to the property of rotation matrix, it exists the following constraint:

(6) $$R^{T}R=I$$

In this way, the computation of the transformation matrix can be transformed into an optimization problem with a constraint:

(7) $$\{\begin{array}{c} \min{{\left\|X_{M}-T_{E-M}X_{E}\right\|}^{2}}_{F}\\ s.t\;R^{T}R=I_{3\times 3} \end{array}$$

By eliminating the translation term, this optimization problem can be converted to an orthogonal-force-consistency problem, which can be solved by single value decomposition.

### 2.4. Global Data Fusion Model

For the structured light profilometry, the data on a surface area is acquired in every scanning. Therefore, to get the entire data of a large-scale component, the measurement should be implemented for a lot of times according to the size. In this process, the scanner is carried to different positions by robot. The surface point cloud data at every position can be obtained. To combine all the data, the position and pose of the end-effector should be tracked. 

For every measurement position, an {*ECS*}*_i_* is built by tracking the CCR ball. Let $T_{E-W}^{i}$ be the transformation between {*ECS*}*_i_* and WCS. $T_{E-W}^{i}$ can be written into the following form:

(8) $$T_{E-W}^{i}=\left(\begin{array}{cccc} n_{x} & o_{x} & p_{x} & t_{x}\\ n_{y} & o_{y} & p_{y} & t_{y}\\ n_{z} & o_{z} & p_{z} & t_{z}\\ 0 & 0 & 0 & 1 \end{array}\right)$$

In this matrix, (*n_x_ n_y_ n_z_*)*^T^*, (*o_x_ o_y_ o_z_*)*^T^*, (*p_x_ p_y_ p_z_*)*^T^* are the corresponding unit vectors of {*ECS*}*_i_* coordinate axes in WCS. (*t_x_ t_y_ t_z_*)*^T^* is the position of the origin point of {*ECS*}*_i_* in WCS. Until now, both the hand-eye transformation matrix *T_E-M_* and the transformation matrix between {*ECS*}*_i_* and WCS ($T_{E-W}^{i}$) have been obtained. To combine all the data, the following equation can be used:

(9) $$T_{M-W}^{i}=T_{E-M}T_{E-W}^{i}$$

Here $T_{M-W}^{i}$ is the transformation matrix between MCS and WCS. With this equation, all the acquired data can be unified into WCS, and the data fusion can be finished automatically.

## 3. Results

To verify the effect of proposed methodologies, several experiments are designed and implemented. Through hand-eye calibration experiment, the transformation matrix between MCS and ECS is computed. Based on this relationship, the global data fusion experiment is executed and the 3D shape of a car body rear is acquired. To the end of quantitative assessment, a metric tool is constructed and the evaluation results are demonstrated.

### 3.1. Hand-Eye Calibration

**Figure 6:**
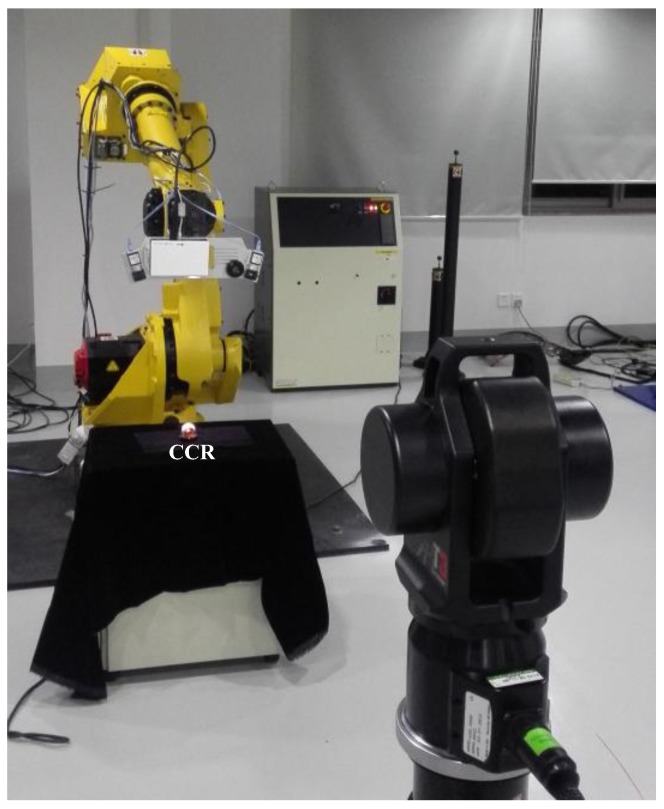
Hand-eye calibration experiment.

The hand-eye calibration algorithm has been introduced in Section 2.2. According to the algorithm, the experiment is designed, which is shown in Figure 6. In the calibration process, the scanner and robot should be kept still. The target ball is put on 15 different positions in the scanner vision field range. For every position, the ball is measured by the scanner and the laser tracker simultaneously. After this, the ball is set on the scanner. After three times’ rotation, the ECS is constructed. The data is shown in Table 1.

With this data, and by using the algorithm proposed in Section 2.2, *T_E_*_−*M*_ is solved finally, which is shown as follows:

(10) $$T_{E-M}=\left[\begin{array}{rrrr} -0.5634 & 0.0051 & -0.8262 & 8.2035\\ 0.8262 & 0.0065 & -0.5633 & -53.9117\\ 0.0025 & -1.0000 & -0.0079 & -11.7849\\ 0.0000 & 0.0000 & 0.0000 & 1.0000 \end{array}\right]$$

### 3.2. Global Data Fusion 

**Figure 7:**
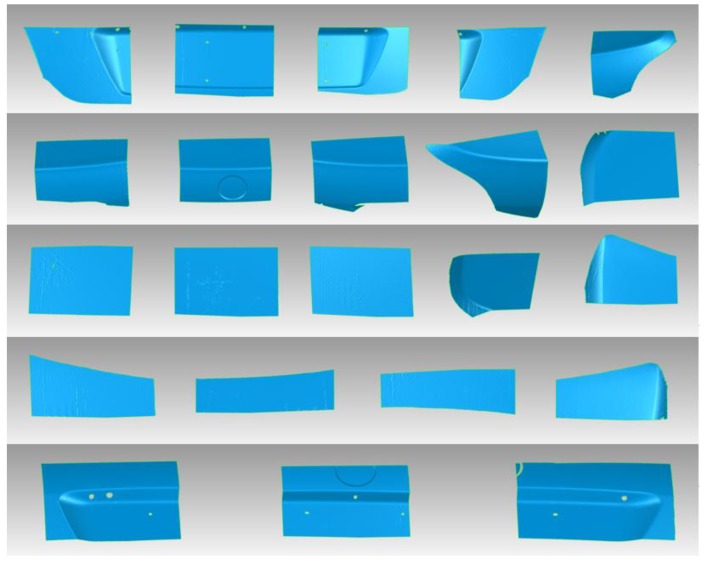
Surface point clouds of 22 times measurement represented in a form of triangular meshes.

After the calibration of the hand-eye relationship, a car body rear with a size of 1400 mm × 500 mm × 400 mm was measured to verify the proposed scheme. The experiment system is illustrated in Figure 3. According to the path planning results, the scanner is carried by the robot to 22 different positions. For every position, corresponding surface data is acquired by the structured light scanner. Figure 7 shows the point cloud data in a form of triangular meshes representation.

**Figure 8:**
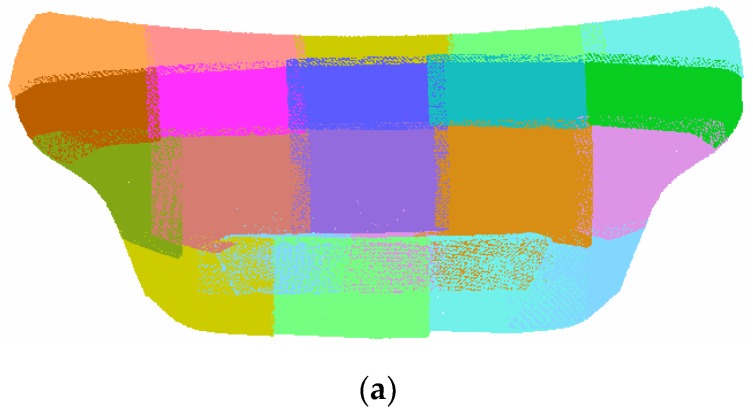

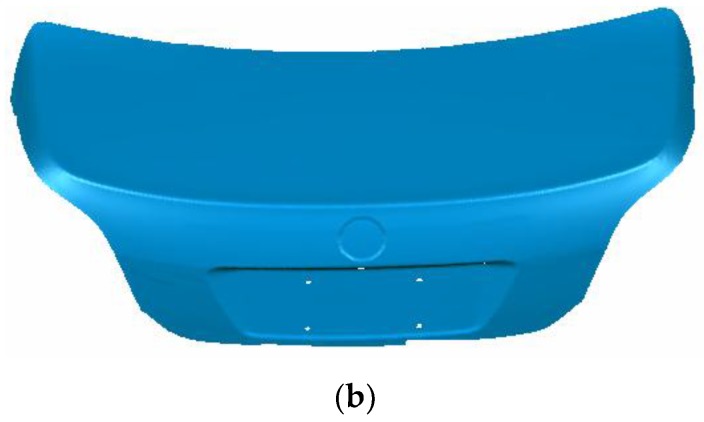
(**a**) Point cloud data fusion result; (**b**) triangular meshes representation.

Simultaneously, {*ECS*}*_i_* is constructed by tracking the CCR ball. According to the method proposed in Section 2.3, $T_{E-W}^{i}$—the transformation matrix between {*ECS*}*_i_* and WCS—is constructed. The hand-eye transformation matrix was presented by Formula (9) in Section 3.1. Therefore, the $T_{M-W}^{i}$ —the transformation matrix between MCS and WCS—can be built according to the algorithm proposed in Section 2.3. With this data, the point cloud data in each position can be transformed into WCS. The global data fusion is implemented automatically. Figure 8 shows the multicolor representation of point cloud data at each measurement position (Figure 8a) and the triangular meshes representation (Figure 8b) of the holistic car rear surface. It is illustrated that there exist overlapping areas between adjacent scans. These overlapping areas have been used to evaluate the fusion accuracy. By a proper path planning, the percentage of the overlapping area is set to 10% to 40%, which is enough for precision computation. In the fusional data, about 20 million points are acquired, which is redundant for accuracy evaluation. Therefore, by a resampling algorithm, the number has been reduced to about 2 million.

A simple visual inspection can be used to assess the alignment accuracy even without proper metric tool. The stripped patterns on the triangular meshes surface represent the misalignment errors between overlapping areas of different point clouds (Figure 8b). 

**Figure 9:**
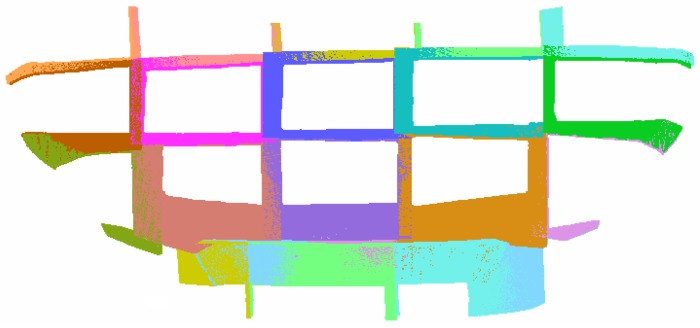
Overlapping areas between all the aligned point clouds.

Although the visual assessment can afford a qualitative evaluation for the alignment precision, the result cannot be considered as exhaustive. To acquire the quantitative result of the misalignment error, the proximity between the overlapping areas (Figure 9) of different point clouds is computed.

A metric tool has been developed to compute the translation and rotation error. Compared to the perpendicular directions (*x* and *y* direction), the misalignment error along the optical scanner viewing direction (*z* direction) dominates [24]. For most of the car rear surface, the curvature is low. Therefore, the error along the z direction is the most significant for alignment precision evaluation.

**Figure 10:**
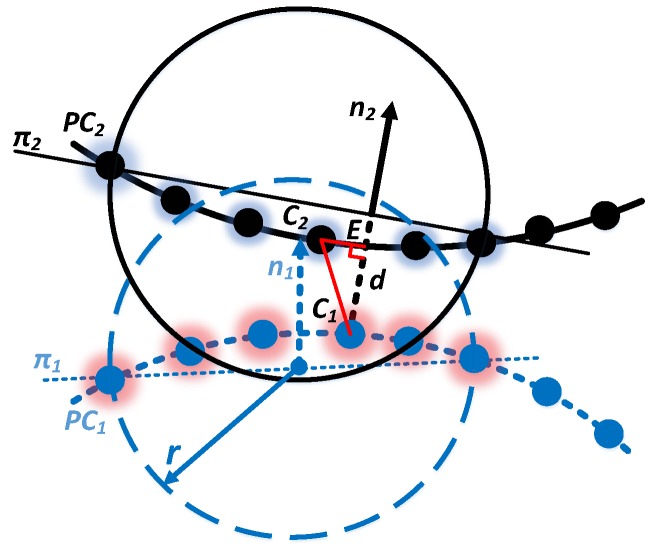
Definition of the distance and angle error.

The translation error is defined as a projection of the distance between the nearest points to the normal vector of the fitting plane. As shown in Figure 10, given two different point clouds (PC1 and PC2), for each point set, the mean distance (dm) between all the points are acquired. Then the distance is used to get a radius which can be used to define a circle. With these points, and by a least-square plane fitting estimation computation, the normal vector (n1, n2) and the best fitting plane (π1, π2) can be computed [27]. Then the nearest point pairs are searched and the distance between these points are acquired. In this way, the distance d from C1 to PC2 is defined as $d=\left|\bar{C_{1}C_{2}}\right|\cos∠EC_{1}C_{2}$.

The rotation error is defined as the angle value between the unit vectors n1 and n2. By traversing all the points in point cloud, the translation and rotation error computation is ultimately finished. As stated in [24], the accuracy of the least square fitting algorithm significantly depends on the radius *r*, which can only be estimated by empirical analyses [28]. In the presented case, the value is defined as *r* = 6 dm.

**Figure 11:**
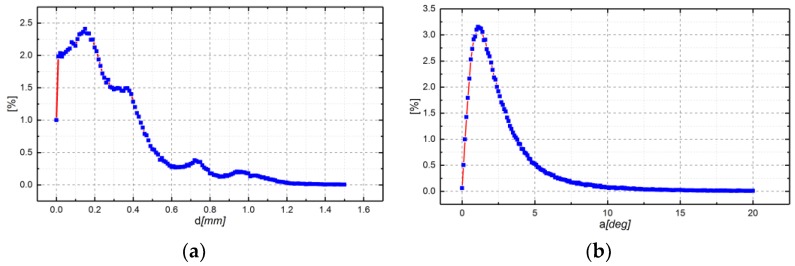
(**a**) Distribution of the translation error; (**b**) distribution of the angle error.

By using the metric tool, the translation and rotation errors are computed, and Figure 11 shows the results. In this Figure, the horizontal axis represents the error value and the vertical axis denotes the percentage of the corresponding error. It is illustrated in Figure 11a that, for most of the points (with a percentage of 88.53%), the distance is less than 0.6 mm. If the threshold value is set to 1 mm, almost all the points (97.76%) are comprised. A similar situation occurs for the rotation error (Figure 11b). Most of the error value is less than 10 degrees (97.21%). To demonstrate the errors more clearly, the maximum value (max), minimum value (min), mean value (µ) and standard deviation value (σ) are also summarized in Table 2. With these quantitative statistic results, the quality of the data fusion can be assessed objectively.

## 4. Conclusions

This paper presents an integrated system for large-scale component profilometry. In this system, a structured light scanner is built to acquire surface point cloud data at each position. The robot is only used as an orienting device in large volume. By establishing the transformation relationship between measurement coordinate system (MCS) and world coordinate system (WCS), all the data is combined into WCS which is defined by laser tracker. For this system, the construction of the end coordinate system (ECS) plays a pivotal role. Here the CCR is mounted on the base of the scanner. After three times’ rotation, the ECS is constructed. Additionally, different from classic hand-eye calibration method, in this scheme, the hand-eye transformation matrix is computed by a synchronized observation of the scanner and laser tracker. This approach makes the hand-eye calibration independent from robot kinematics parameters, which makes the calibration more robust and easier to be implemented. An algorithm is also built to solve the transformation matrix between ECS and WCS. In this way, all the data can be automatically combined to the unified coordinate system. To verify the effect of the proposed method, corresponding experiments are designed and conducted. With this data, the transformation relationship between MCS and WCS is computed. Finally, all the data is combined into the same coordinate system, and the shape of a car body rear is reconstructed successfully. To evaluate the precision of the proposed method, a metric tool is developed and the accuracy data is presented. The translation error is less than 0.6 mm for most of the points (88.53%). A mean/maximum value of 0.2965/1.5081 mm is detected in the work volume. The standard deviation is 0.2465 mm. For rotation error, the mean and maximum value are 2.8333 and 20.0841 degrees respectively. The standard deviation of the rotation error is 2.6185 degrees. 

The mean value and standard deviation demonstrate that the integrated system exhibits good accuracy which is comparable to accuracy of the existing system [16,24]. It is believed that the proposed scheme is of relatively high-efficiency and easy to be implemented. It is quite suitable for the measurement of large-scale components, such as car bodies, ship plates and astronautical/aeronautical large-scale thin wall components. Future work will focus on more intelligent path planning algorithm and the improvement of measuring accuracy.

## Figures and Tables

**Table 1:** Hand-eye calibration data.

	WCS			ECS			MCS		
	X	Y	Z	X	Y	Z	X	Y	Z
No.1	−603.234	−570.824	−623.064	−654.862	−154.646	−104.806	289.155	88.323	605.415
No.2	−527.529	−541.327	−623.513	−667.577	−167.702	−25.628	285.935	9.639	622.489
No.3	−440.814	−505.516	−621.711	−679.309	−183.062	66.197	280.02	−81.623	639.968
No.4	−456.599	−455.086	−621.325	−649.743	−226.843	67.418	227.691	−83.119	640.253
No.5	−540.046	−475.961	−622.876	−631.899	−223.167	−16.662	220.646	0.517	624.339
No.6	−634.259	−513.428	−622.370	−616.476	−206.324	−115.448	225.462	98.891	602.866
No.7	−657.153	−469.599	−621.906	−587.494	−245.701	−122.851	176.813	106.193	601.124
No.8	−581.189	−435.511	−622.558	−598.298	−262.613	−42.041	169.253	25.723	618.982
No.9	−476.216	−395.233	−620.951	−614.414	−278.986	68.033	165.075	49.782	551.496
No.10	−494.938	−344.801	−620.797	−584.017	−323.345	66.487	111.551	−82.539	640.278
No.11	−577.894	−369.441	−621.975	−567.816	−316.284	−18.234	107.993	1.811	623.791
No.12	−671.393	−671.393	−621.606	−549.986	−304.248	−114.556	107.749	97.758	603.093
No.13	−690.162	−353.111	−621.409	−520.717	−346.523	−116.918	56.707	99.861	602.826
No.14	−618.261	−325.513	−621.534	−532.745	−358.414	−41.782	53.710	25.136	618.746
No.15	−513.567	−284.161	−620.443	−548.676	−375.996	68.258	48.626	−84.303	640.652

**Table 2:** Statistic of the distance and angle error.

	max	min	μ	σ
d (mm)	1.5081	0	0.2965	0.2465
A (deg.)	20.0841	0.0029	2.8333	2.6185
